# Tumor in Anterior Leaflet of the Mitral Valve

**DOI:** 10.21470/1678-9741-2020-0557

**Published:** 2021

**Authors:** Francielle Santos Almeida, Gabriele Justo Canevazzi, Priscila Barão Rocha, Ana Cristina Carlomagno Molinari Sobral, Marcelo Luiz Peixoto Sobral

**Affiliations:** 1 Faculdade de Medicina, Centro Universitário das Américas, São Paulo, São Paulo, Brazil.; 2 Echocardiography Department, Hospital Beneficência Portuguesa de São Paulo, São Paulo, São Paulo, Brazil.; 3 Departament of Cardiovascular Surgery, Hospital Beneficência Portuguesa de São Paulo, São Paulo, São Paulo, Brazil.; 4 Post Graduate Department of Cardiovascular and Thoracic Surgery, Universidade de São Paulo, Instituto do Coração, São Paulo, São Paulo, Brazil.

**Table:** 

Abbreviations, acronyms & symbols	
3D	= Three-dimensional
AO	= Aorta
CM	= Cardiac myxoma
LA	= Left atrium
LV	= Left ventricle
LVOT	= Left ventricular outflow tract
TEE	= Transesophageal echocardiogram
TTE	= Transthoracic echocardiography

## INTRODUCTION

**Fig. 1:**
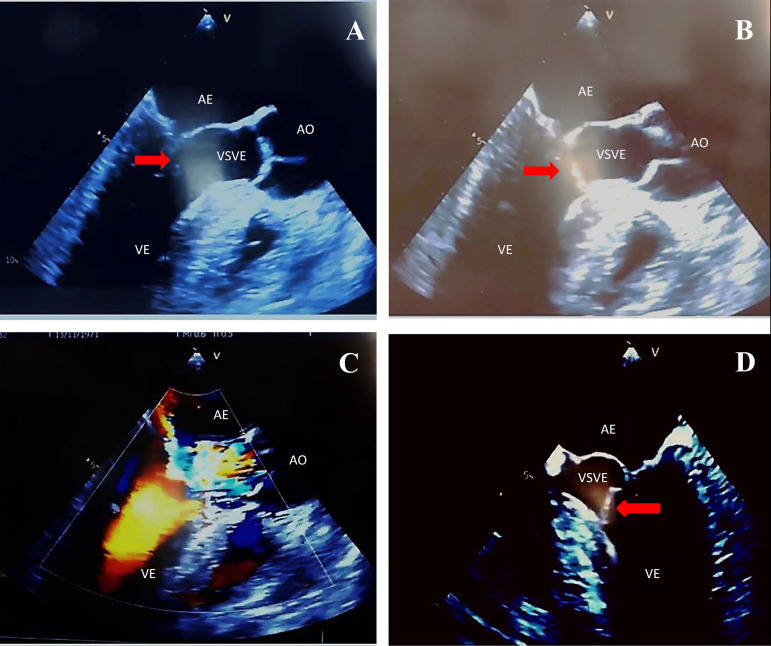
(A) Mitral valve partially closed with hyperechogenic image (red arrow) adhered to its anterior leaflet (esophageal image with long axis at 120 degrees); (B) mitral valve during ventricular systole with a hyperechogenic image (red arrow) adhered to its anterior leaflet, partially obstructing the LVOT (120-degree long-axis esophageal image); (C) acceleration of systolic flow due to partial obstruction of the LVOT (long-axis esophageal image at 120 degrees); (D) hyperechogenic image (red arrow) adhered to the anterior mitral valve cusp partially obstructing LVOT in systole (esophageal image of 4 chambers at 0 degrees). AO=aorta; LA=left atrium; LV=left ventricle; LVOT=left ventricular outflow tract

**Fig. 2:**
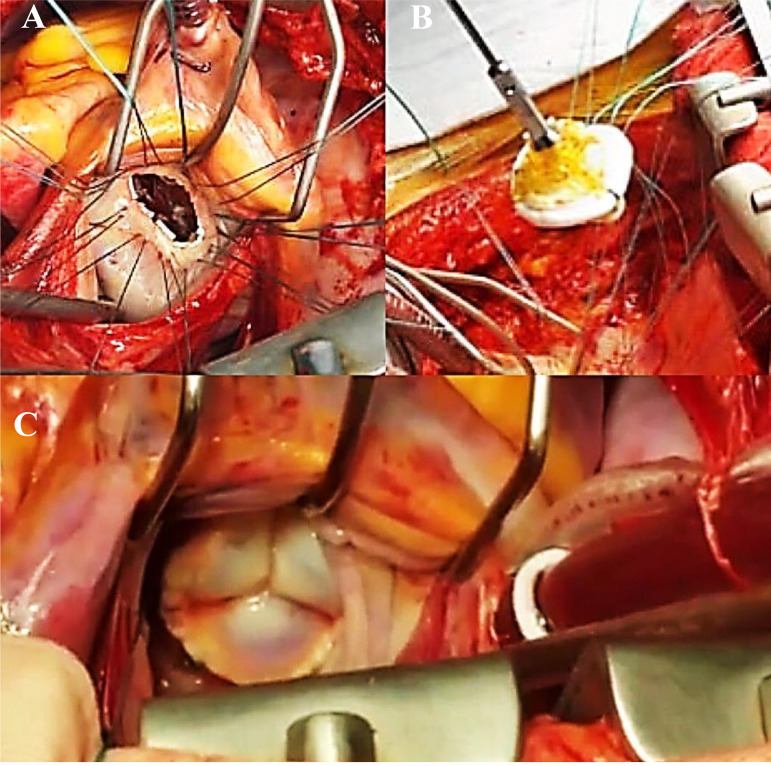
(A) Mitral ring exposed by the left atriotomy, separate vertical "U" suture to fix the bioprosthesis; (B) wires passed through the bioprosthesis; (C) final aspect of the bioprosthesis fixed in the correct position.

**Fig. 3:**
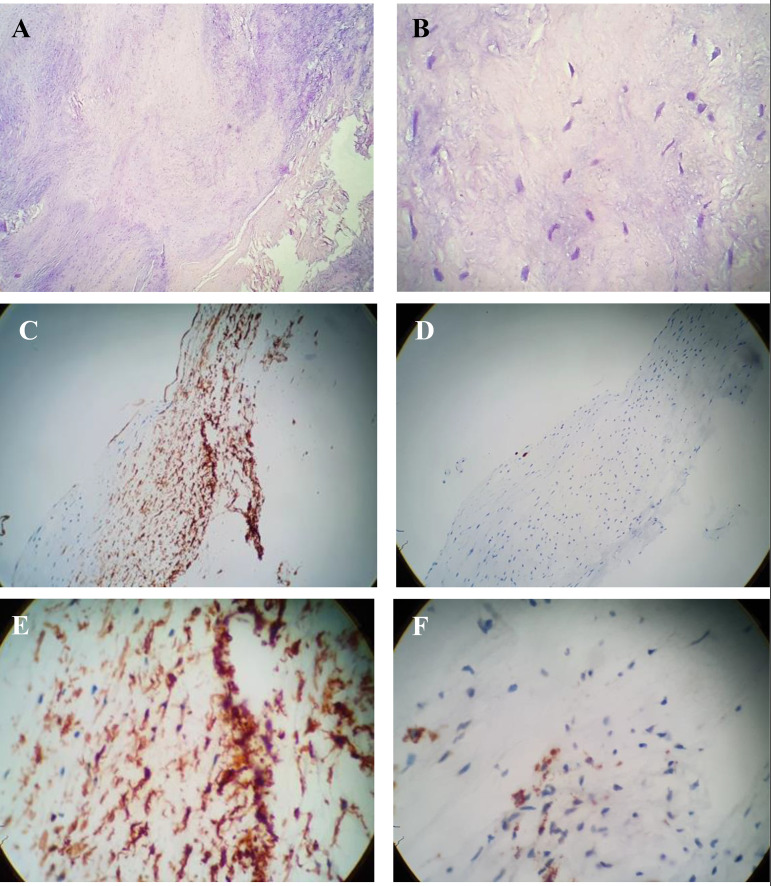
Anatomopathological study (A, 4× magnification; B, 40× magnification) - Chronic degenerative valve disease, at the expense of hyalinized fibrosis and myxoid degeneration of the stroma. *Fibrosis (pink color); myxoid degeneration of the valve stroma (blue color). Immunohistochemistry of valve tissue (C, D, E, and F) - chronic valve disease with fibrosis and stromal myxoid disorder. *CD3, rare T lymphocytes; CD20, rare B lymphocytes; CD34, stromal positivity; CD68, negative; Ki-67 antigen, low index of cell proliferation.

This is the case of a 47-year-old male who developed mild dyspnea on great exertion. Routine transthoracic echocardiography (TTE) revealed a slightly thickened mitral valve with anterior leaflet prolapsed and mild incompetence. There was an echogenic image suggestive of an irregular and mobile tumor adhered to the ventricular face of the anterior mitral valve leaflet, measuring about 17 mm by 10 mm. The movement of the tumor contributed to a dynamic increase in the left ventricular outflow gradient, estimated at 33 mmHg. The transesophageal echocardiogram (TEE) performed 14 days later showed prolapse of the anterior mitral valve leaflet, which presents a movable filamentary mass, measuring 13 mm by 3 mm, located at its end on the ventricular surface, and which protruded during systole via the left ventricular outlet, causing dynamic obstruction with peak systolic gradient of 59 mmHg. Doppler revealed mild to moderate reflux (Figure 1). Surgery for mitral replacement or repair with tumor resection was performed with replacement of the mitral valve by bioprosthesis 29 (Figure 2). The fragment of the anterior leaflet was submitted to histological examination and immunohistochemical study (Figure 3).

## QUESTIONS

- What are the diagnostic possibilities and their characteristics?
- Regarding imaging techniques, were they the most suitable for diagnosis?
- What was decisive for surgical treatment? Does the surgical indication depend on the presence of symptoms?
- Is there a need for anticoagulation and association of arrhythmias in this type of tumor?

### Discussion of Questions

QUESTION A. The main diagnostic possibilities would be cardiac myxoma (CM), chordae rupture, papillary fibroelastoma, vegetation, and the redundant cusp itself. CM should be suspected in patients with chronic dyspnea, history of peripheral or systemic embolization, acute neurological disorder, and family history of CM^[1]^. However, approximately 3.2% to 46.4% of patients with CM are asymptomatic^[2-6]^. CM is a rare benign cardiac tumor, and its incidence is observed in approximately 0.5-1 case per 1 million people per year^[7-10]^. Among primary cardiac tumors, myxoma is responsible for 50% of cases^[6]^, whose average age range varies from 42 to 66 years^[11,12]^. 

QUESTION B. The echocardiogram is the main modality of diagnostic imaging in the morphological evaluation of intracardiac tumors. TTE is defined as a first-line imaging modality for assessing valve anatomy, severity of mitral regurgitation, hemodynamics, and ventricular consequences. When TTE does not add value to the diagnosis, TEE is always advocated as an enhancement for additional diagnosis^[13]^. However, the three-dimensional (3D) echocardiogram provides a better characterization and morphological classification according to the microscopic appearance of the surface of these intracardiac masses, with good correlation with surgical and histopathological findings^[13]^, but the need for greater training of operators, cost, and high dependence on image quality currently limit the wide application of 3D in the oncology scenario^[14]^. Studies have shown that two-dimensional and 3D techniques are equally reliable, and that 3D TEE has an advantage in locating the disease^[15-18]^. 

QUESTION C. For symptomatic patients, surgical exeresis is the treatment of choice, always trying to preserve the valve tissue and its function. In asymptomatic individuals, surgical management is controversial, with tumor mobility being the determining factor for surgical indication, as it is an independent predictor of embolization and death^[19-21]^. Due to malignant potentials, timely surgical resection is guaranteed in all patients, without contraindication when making the diagnosis, as the tumor's behavior cannot be predicted and does not depend on how large and fragile it is.

QUESTION D. The follow-up of asymptomatic patients who do not undergo surgery should include echocardiographic monitoring and anticoagulation therapy, although its effectiveness in protecting against embolic phenomena is controversial^[22]^. Embolization is more likely to occur in mobile papillary cardiac tumors, which are smaller in size, and in patients who have preoperative atrial fibrillation, and it is necessary to treat embolic events with anticoagulation to avoid serious events such as stroke, an embolism in the peripheral vasculature, or an embolism in the pulmonary artery, caused by detached tumor tissue or mobilization of thrombotic deposits. Usually the occurrence of arrhythmias, mainly extrasystoles and atrial fibrillation, is due to some obstruction of the physiological blood flow^[23,24]^.

## BRIEF CONSIDERATION OF THE CASE REPORTED

Primary cardiac tumors are rare, with a prevalence between 0.0017% and 0.19% of the unselected autopsy studies. About 75% are benign tumors and almost half are myxomas. The rest are divided among rhabdomyomas, lipomas, and fibroelastomas.

Due to its predominant involvement in cardiac valves, nonspecific symptoms, and characteristics of its shape and size, the chordae rupture, papillary fibroelastoma, vegetation, and the redundant cusp itself have become suggestive in this clinical case.

It is important to note that obstruction of the heart valve caused by CM, for example, can lead to catastrophic consequences, including sudden death. A fragment of the tumor can embolize to the left or right coronary artery ostium and result in acute coronary syndrome, and surgical resection has a low rate of complications and mortality. This detail was also important for the decision for surgical treatment.

**Table:** 

Authors' roles & responsibilities	
FSA	Substantial contributions to the conception or design of the work and the acquisition, analysis, or interpretation of data for the work; drafting the work and revising it critically for important intellectual content; final approval of the version to be published
GJC	Substantial contributions to the conception or design of the work and the acquisition, analysis, or interpretation of data for the work; drafting the work and revising it critically for important intellectual content; final approval of the version to be published
PBR	Substantial contributions to the conception or design of the work and the acquisition, analysis, or interpretation of data for the work; drafting the work and revising it critically for important intellectual content; final approval of the version to be published
ACCMS	Substantial contributions to the conception or design of the work and the acquisition, analysis, or interpretation of data for the work; drafting the work and revising it critically for important intellectual content; final approval of the version to be published
MLPS	Substantial contributions to the conception or design of the work and the acquisition, analysis, or interpretation of data for the work; drafting the work and revising it critically for important intellectual content; final approval of the version to be published

## References

[1] Samanidis G, Khoury M, Balanika M, Perrea DN (2020). Current challenges in the diagnosis and treatment of cardiac myxoma. Kardiol Pol.

[2] He DK, Zhang YF, Liang Y, Ye SX, Wang C, Kang B (2015). Risk factors for embolism in cardiac myxoma a retrospective analysis. Med Sci Monit.

[3] Patil NP, Dutta N, Satyarthy S, Geelani MA, Kumar Satsangi D, Banerjee A (2011). Cardiac myxomas experience over one decade. J Card Surg.

[4] Lee SJ, Kim JH, Na CY, Oh SS (2012). Eleven years' experience with Korean cardiac myxoma patients focus on embolic complications. Cerebrovasc Dis.

[5] Lee KS, Kim GS, Jung Y, Jeong IS, Na KJ, Oh BS (2017). Surgical resection of cardiac myxoma-a 30-year single institutional experience. J Cardiothorac Surg.

[6] Karabinis A, Samanidis G, Khoury M, Stavridis G, Perreas K (2018). Clinical presentation and treatment of cardiac myxoma in 153 patients. Medicine (Baltimore).

[7] Pinede L, Duhaut P, Loire R (2001). Clinical presentation of left atrial cardiac myxoma A series of 112 consecutive cases. Medicine (Baltimore).

[8] Wu X, Yang D, Yang Z, Li J, Zhao Y, Wang K (2012). Clinical characteristics and long term post-operative outcome of cardiac myxoma. EXCLI J.

[9] Jiang CX, Wang JG, Qi RD, Wang W, Gao LJ, Zhao JH (2019). Long-term outcome of patients with atrial myxoma after surgical intervention analysis of 403 cases. J Geriatr Cardiol.

[10] Yuan SM (2014). Cerebral infarction due to cardiogenic emboli originating from atrial myxoma: a case report. Changhua J Med.

[11] Bianchi G, Margaryan R, Kallushi E, Cerillo AG, Farneti PA, Pucci A (2019). Outcomes of video-assisted minimally invasive cardiac myxoma resection. Heart Lung Circ.

[12] Abu Abeeleh M, Saleh S, Alhaddad E, Alsmady M, Alshehabat M, Bani Ismail Z (2017). Cardiac myxoma clinical characteristics, surgical intervention, intra-operative challenges and outcome. Perfusion.

[13] Lancellotti P, Tribouilloy C, Hagendorff A, Popescu BA, Edvardsen T, Pierard LA (2013). Recommendations for the echocardiographic assessment of native valvular regurgitation: an executive summary from the European association of cardiovascular imaging. Eur Heart J Cardiovasc Imaging.

[14] Garatti A, Nano G, Canziani A, Gagliardotto P, Mossuto E, Frigiola A (2012). Surgical excision of cardiac myxomas: twenty years experience at a single institution. Ann Thorac Surg.

[15] Stewart WJ, Griffin B, Thomas JD (1995). Multiplane transesophageal echocardiographic evaluation of mitral valve disease. Am J Card Imaging.

[16] Lambert AS, Miller JP, Merrick SH, Schiller NB, Foster E, Muhiudeen-Russell I (1999). Improved evaluation of the location and mechanism of mitral valve regurgitation with a systematic transesophageal echocardiography examination. Anesth Analg.

[17] Pepi M, Tamborini G, Maltagliati A, Galli CA, Sisillo E, Salvi L (2006). Head-to-head comparison of two- and three-dimensional transthoracic and transesophageal echocardiography in the localization of mitral valve prolapse. J Am Coll Cardiol.

[18] Hien M, Rauch H, Lichtenberg A, De Simone R, Weimer M, Ponta OA, Rosendal C (2013). Real-time three-dimensional transesophageal echocardiography: improvements in intraoperative mitral valve imaging. Anesth Analg.

[19] Hien MD, Rauch H, Lichtenberg A, De Simone R, Weimer M, Ponta OA (2013). Real-time three-dimensional transesophageal echocardiography: improvements in intraoperative mitral valve imaging. Anesth Analg.

[20] Gowda RM, Khan IA, Nair CK, Mehta NJ, Vasavada BC, Sacchi TJ (2003). Cardiac papillary fibroelastoma: a comprehensive analysis of 725 cases. Am Heart J.

[21] Di Mattia DG, Assaghi A, Mangini A, Ravagnan S, Bonetto S, Fundarò P (1999). Mitral valve repair for anterior leaflet papillary fibroelastoma: two case descriptions and a literature review. Eur J Cardiothorac Surg.

[22] Sun JP, Asher CR, Yang XS, Cheng GG, Scalia GM, Massed AG (2001). Clinical and echocardiographic characteristics of papillary fibroelastomas: a retrospective and prospective study in 162 patients. Circulation.

[23] Vizzardi E, Faggiano P, Antonioli E, Zanini G, Chiari E, Nodari S (2009). Thrombus or tumor? a case of fibroelastoma as indicated during the submission process. Cases J.

[24] Nishimura RA, Otto CM, Bonow RO, Carabello BA, Erwin JP 3rd, Guyton RA (2014). 2014 AHA/ACC guideline for the management of patients with valvular heart disease: executive summary: a report of the American college of cardiology/American heart association task force on practice guidelines. Circulation.

